# Coverage and Prior Authorization Policies for Semaglutide and Tirzepatide in Medicare Part D Plans

**DOI:** 10.1001/jamanetworkopen.2025.29842

**Published:** 2025-08-29

**Authors:** Xiaoyu Liu, Chuan Angel Lu, Ya-Chen Tina Shih, Changchuan Jiang

**Affiliations:** 1 Department of Health Policy & Management, UCLA Fielding School of Public Health, Los Angeles, California; 2 Division of Hematology and Oncology, Department of Internal Medicine, University of Texas Southwestern Medical Center, Dallas; 3 Program in Cancer Health Economics Research, Jonsson Comprehensive Cancer Center, and Department of Radiation Oncology, School of Medicine, University of California, Los Angeles; 4 O’Donnell School of Public Health, University of Texas Southwestern Medical Center, Dallas

## Abstract

This cross-sectional study examines trends in coverage of glucagon-like peptide-1 receptor agonists, semaglutide and tirzepatide, and prior authorization requirements among Medicare Part D beneficiaries.

## Introduction

Glucagon-like peptide-1 (GLP-1) receptor agonists, together with dual glucose-dependent insulinotropic polypeptide (GIP)/GLP-1 agents, belong to the broader class of incretin receptor agonists and are effective therapies for type 2 diabetes, cardiovascular disease (CVD) prevention, and weight management.^1,2^ As the Centers for Medicare & Medicaid Services (CMS) considers coverage for obesity-related use, approximately 19% of Medicare beneficiaries (approximately 13 million individuals) could already benefit from these therapies for non-weight loss indications, such as diabetes and CVD.^3^ Medicare spending on these drugs rose from $57 million in 2018 to $5.7 billion in 2022,^4^ prompting cost-containment efforts by Part D plans, including the use of prior authorization (PA). This study examines trends in coverage and PA requirements for 3 GLP-1–based therapies with the highest prescribing volume: injectable semaglutide (Ozempic, a selective GLP-1 receptor agonist), injectable tirzepatide (Mounjaro, a dual GIP/GLP-1 receptor agonist), and oral semaglutide (Rybelsus, a selective GLP-1 receptor agonist).

## Methods

We used quarterly CMS Basic Drugs Formulary File data from 2020 quarter (Q) 2 to 2024 Q3, covering all Medicare Advantage Part D (MAPD) and standalone prescription drug plans (PDPs). Trends in formulary coverage and PA requirements for the 3 therapies were analyzed and compared across plan types using a 2-sided Wilcoxon signed-rank test, with a statistical significance level of *P* < .05. All analyses were weighted by quarterly enrollment (eMethods in Supplement 1). Data were analyzed using SAS, version 9.4 (SAS Institute). The study was exempt from review by the University of Texas Southwestern Human Research Protection Program because it was deemed nonhuman research. This cross-sectional study followed the Strengthening the Reporting of Observational Studies in Epidemiology (STROBE) guidelines.

## Results

**Figure 1.**
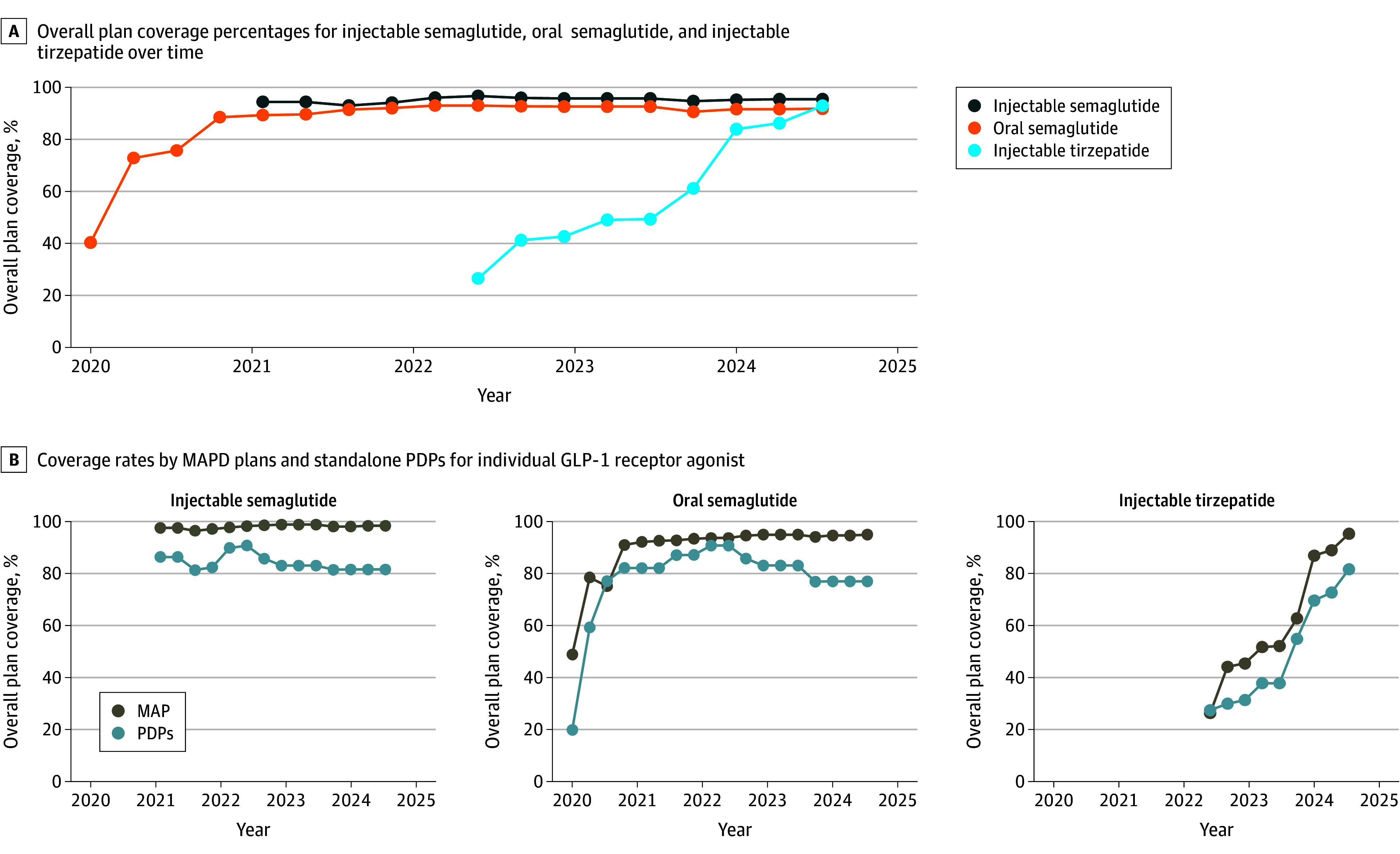
Formulary Coverage Trends for Glucagon-Like Peptide-1 (GLP-1) Receptor Agonists in Medicare Part D Plans, 2020–2024 The figure displays the earliest quarter in which each drug first appeared on Medicare Part D formularies within our dataset, which might not align with the US Food and Drug Administration (FDA) approval dates. For instance, injectable semaglutide was approved for type 2 diabetes on December 5, 2017, and for cardiovascular risk reduction on January 16, 2020; however, it did not appear on any plan formulary in our data until 2021 quarter (Q)2. Similarly, oral semaglutide, approved on September 20, 2019, was not observed in coverage data until 2021 Q2. In contrast, injectable tirzepatide, approved on May 13, 2022, first appeared in our dataset by 2022 Q3. These discrepancies reflect the lag between FDA approval and formulary adoption, rather than inconsistencies in regulatory timelines. MAPD, indicates Medicare Advantage Prescription Drug; PDPs, prescription drug plans; Q, quarter.

Our analysis included 54 358 MAPD and 15 895 PDP quarter-plan observations. Coverage for injectable semaglutide remained above 90% since 2021. Injectable tirzepatide coverage increased from 26.5% in 2022 Q3 to 92.9% in 2024 Q3, and oral semaglutide from 40.3% in 2020 Q3 to 91.8% in 2024 Q3. Coverage rates were consistently higher among MAPDs than PDPs (*P* < .01) (Figure 1).

**Figure 2.**
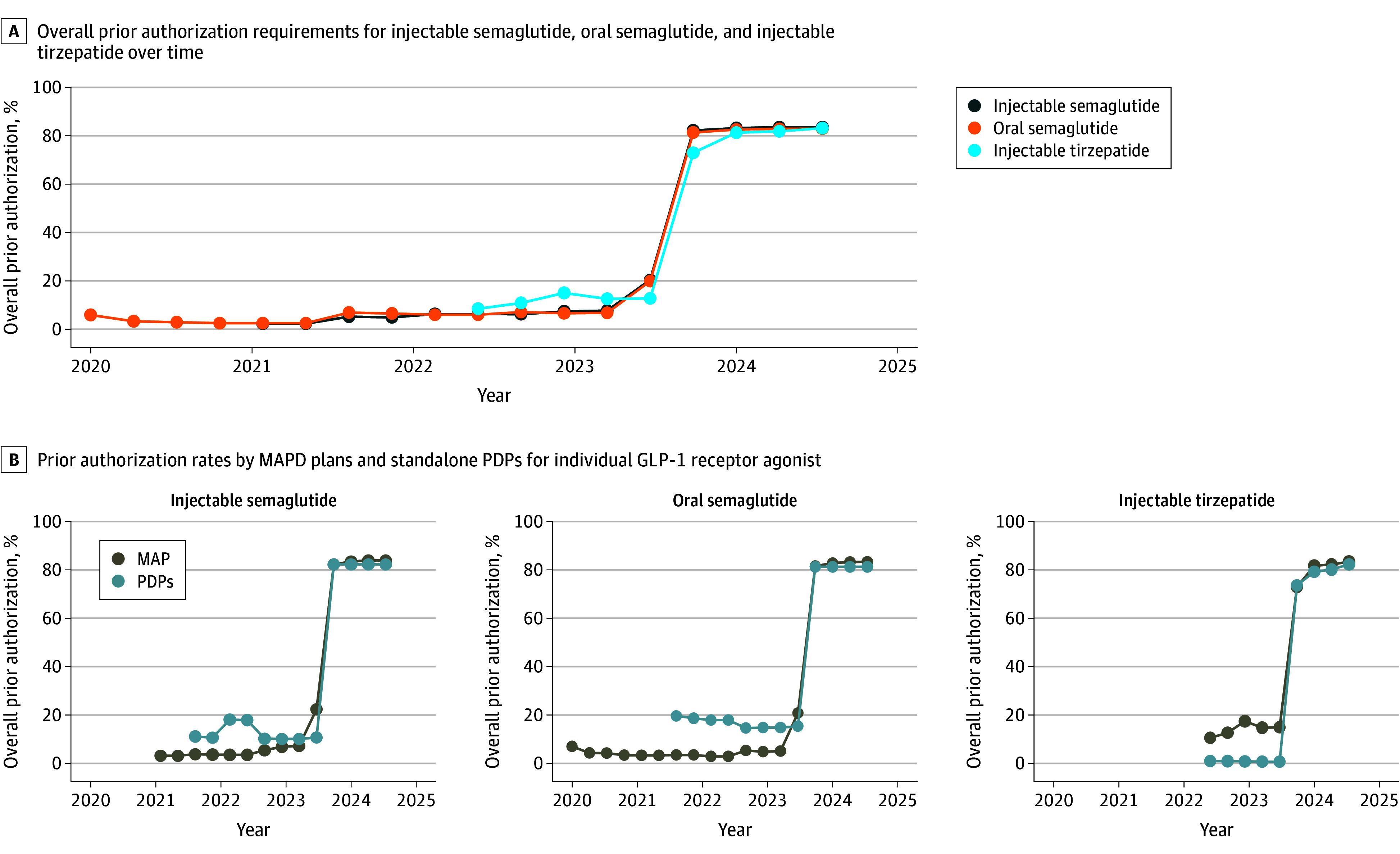
Trends in Prior Authorization for Glucagon-Like Peptide-1 (GLP-1) Receptor Agonists in Medicare Part D Plans, 2020–2024 The figure displays the earliest quarter in which each drug first appeared on Medicare Part D formularies within our dataset, which might not align with the US Food and Drug Administration (FDA) approval dates. For instance, injectable semaglutide was approved for type 2 diabetes on December 5, 2017, and for cardiovascular risk reduction on January 16, 2020; however, it did not appear on any plan formulary in our data until 2021 quarter (Q)2. Similarly, oral semaglutide, approved on September 20, 2019, was not observed in coverage data until 2021 Q2. In contrast, injectable tirzepatide, approved on May 13, 2022, first appeared in our dataset by 2022 Q3. These discrepancies reflect the lag between FDA approval and formulary adoption, rather than inconsistencies in regulatory timelines. MAPD indicates Medicare Advantage Prescription Drug; PDPs, prescription drug plans; Q, quarter.

Among plans that covered these therapies, PA requirements remained below 25% until 2023 Q3, then rose sharply to 83.6%, 83.2%, and 83.0% in 2024 Q3 for injectable semaglutide, tirzepatide, and oral semaglutide, respectively. After this increase, PA rates were similar across MAPDs and PDPs (Figure 2). No variation in coverage or PA was observed across dose levels.

## Discussion

Our analysis revealed a substantial expansion in coverage for injectable semaglutide, injectable tirzepatide, and oral semaglutide from 2020 to 2024, accompanied by a sharp rise in PA requirements, from below 15% in 2023 Q3 to over 80% in 2024 Q3. Although coverage for these GLP-1 receptor agonists remained consistently higher among MAPDs than standalone PDPs, both plan types rapidly adopted PA requirements beginning in late 2023.

The widespread adoption of PA likely reflects efforts to manage costs amid expanding clinical indications and growing demand. Although PA serves a legitimate gatekeeping role—ensuring appropriate prescribing and protecting drug supply—the sudden scale-up raises concerns about its clinical consequences. The administrative burden associated with PA might disproportionately affect underresourced physicians, particularly those serving underserved populations.^5^ Even among patients with formal drug coverage, these barriers could delay access and widen disparities,^6^ particularly salient for Medicare beneficiaries, who are older, medically complex, and at elevated cardiometabolic risk. Timely access to GLP-1 receptor agonists is essential for this population, and rising administrative demands might unintentionally deepen inequities in care.

Study limitations include the lack of patient-level data and the inability to assess PA outcomes or plan-specific approval patterns. Future work should consider linking claims or prescriber data to enrich our understanding of denial rates, approval timelines, actual access, physician prescribing behavior, and patient adherence, as well as conducting plan- and market-level analysis to explore heterogeneity among PA policies. To ensure formulary coverage translates into clinical benefit, policymakers should prioritize equity-focused PA reforms, such as enhancing transparency, monitoring disparities, and supporting physicians in underresourced settings.

## Notes

Supplement 1.
eMethods

Supplement 2.
Data Sharing Statement
