# Supplementary material for: Coverage and Prior Authorization Policies for Semaglutide and Tirzepatide in Medicare Part D Plans
Source: JAMA Netw Open. 2025 Aug 29;8(8):e2529842. doi: 10.1001/jamanetworkopen.2025.29842 (PMC12397888; doi:10.1001/jamanetworkopen.2025.29842)
Supplement: Supplement 1. — eMethods [file jamanetwopen-e2529842-s001.pdf]

Supplemental Online Content

Liu X, Lu CA, Shih Y-CT, Jiang C. Coverage and prior authorization policies for semaglutide and tirzepatide in Medicare Part D plans. *JAMA Netw Open.* 2025;8(9):e2529842. doi:10.1001/jamanetworkopen.2025.29842

eMethods.

This supplemental material has been provided by the authors to give readers additional information about their work.

© 2025 Liu X et al. *JAMA Network Open*.

eMethods.

Data source:

1. Quarterly Prescription Drug Plan Formulary, Pharmacy Network, and Pricing Information | CMS Data. Available at: https://data.cms.gov/provider-summary-by-type-of-service/medicare-part-d-prescribers/quarterly-prescription-drug-plan-formulary-pharmacy-network-and-pricing-information
2. Medicare Advantage/Part D Contract and Enrollment Data | CMS. Available at: https://www.cms.gov/data-research/statistics-trends-and-reports/medicare-advantagepart-d-contract-and-enrollment-data
3. Monthly Enrollment by Plan | CMS. Accessed June 13, 2025. https://www.cms.gov/data-research/statistics-trends-and-reports/medicare-advantagepart-d-contract-and-enrollment-data/monthly-enrollment-plan

Main Dataset:

The Quarterly Centers for Medicare & Medicaid Services (CMS) Basic Drugs Formulary File from 2020Q2 to 2024Q3, which includes classified Part D plan types for Medicare Advantage prescription drug plans (MAPDs) and independent prescription drug plans (PDPs) (data source #1, #2).

Medicare Plan and Drug Selection:

To focus on the plans most widely available to the public, we included Health Maintenance Organization (HMO), Health Maintenance Organization – Point of Service (HMO-POS), and Preferred Provider Organization (PPO) plans. We excluded employer-sponsored and Special Needs Plans (including those for dual-eligible individuals), as well as plans with fewer than 10 beneficiaries based on CMS plan enrollment data from September 2024.

We identified FDA-approved, non-obesity-indicated glucagon-like peptide-1 (GLP-1) receptor agonists using their National Drug Codes (NDCs) and the National Library of Medicine's RxNorm Concept Unique Identifiers (RXCUIs) in each quarterly formulary file (data source #1).

Coverage and Prior Authorization Rate Estimates:

Medicare plan coverage rates were calculated based on the proportion of selected GLP-1 drugs among total plan offerings, stratified by PDP and MAPD plan types for each quarterly file. Among the covered GLP-1 drugs in PDP and MAPD plans, prior authorization (PA) rates were summarized according to the PA status, documented as a binary variable for each unique formulary ID. To ensure accurate representation of the Medicare beneficiary population, all estimates were weighted using plan-level enrollment data on given year-quarter (data source #3), with larger plans contributing proportionally greater weight to the plan coverage and PA rate calculations.

Data Visualizations:

Since Medicare plan coverage and PA rates for each selected GLP-1 drug did not vary by dosage, as differentiated by RXCUI identifiers in both MAPDs and PDPs, estimates were visualized based on the generic drug type and corresponding brand name. Trend visualizations for coverage and PA rates over the study period were created using Microsoft Excel (Version 16.93.1).

© 2025 Liu X et al. *JAMA Network Open*.
