# Supplementary material for: Coverage and Prior Authorization Policies for Semaglutide and Tirzepatide in Medicare Part D Plans
Source: JAMA Netw Open. 2025 Aug 29;8(8):e2529842. doi: 10.1001/jamanetworkopen.2025.29842 (PMC12397888; doi:10.1001/jamanetworkopen.2025.29842)
Supplement: Supplement 2. — Data Sharing Statement [file jamanetwopen-e2529842-s002.pdf]

# Data Sharing Statement

Liu. Coverage and Prior Authorization Policies for Semaglutide and Tirzepatide in Medicare Part D Plans. *JAMA Netw Open*. Published September 02, 2025. doi:10.1001/jamanetworkopen.2025.29842

**Data**
**Data available:** No

**Additional Information**
**Explanation for why data not available:** As the data are publicly available through CMS, no special permissions or access restrictions apply. Researchers can access and use the same data for replication or further analysis by visiting the CMS website and downloading the relevant datasets. The authors did not generate or collect original data for this study.
